# Multicenter Phase II Trial of the PARP Inhibitor Olaparib in Recurrent *IDH1-* and *IDH2*-mutant Glioma

**DOI:** 10.1158/2767-9764.CRC-22-0436

**Published:** 2023-02-02

**Authors:** Kristina Fanucci, Mary Jo Pilat, Derek Shyr, Yu Shyr, Scott Boerner, Jing Li, Diane Durecki, Jan Drappatz, Vinay Puduvalli, Frank Scott Lieberman, Javier Gonzalez, Pierre Giglio, S. Percy Ivy, Ranjit S. Bindra, Antonio Omuro, Patricia LoRusso

**Affiliations:** 1 Yale Cancer Center, New Haven, Connecticut.; 2 Wayne State University, Detroit, Michigan.; 3 Department of Biostatistics, Harvard T.H. Chan School of Public Health, Boston, Massachusetts.; 4 Department of Biomedical Informatics, Vanderbilt University, Nashville, Tennessee.; 5 University of Michigan Medical Center, Ann Arbor, Michigan.; 6 University of Pittsburgh Cancer Institute, Pittsburgh, Pennsylvania.; 7 Division of Neuro-Oncology, The Ohio State University Wexner Medical Center, Columbus, Ohio.; 8 NCI, Bethesda, Maryland.

## Abstract

**Purpose:**

Isocitrate dehydrogenase (*IDH*) *1* and *IDH2* mutations (*IDH1/2*mt) are frequent in glioma. Preclinical studies suggest *IDH1/2*mts confer “BRCAness” phenotype, a vulnerability that can be targeted through PARP inhibition. To test this hypothesis, we conducted a multicenter study of olaparib monotherapy in patients with *IDH1/2*mt gliomas.

**Methods:**

Patients with recurrent, contrast-enhancing *IDH1/2*mt gliomas were enrolled in a two-step phase II trial; the primary endpoint was overall response rate per Response Assessment in Neuro-Oncology (RANO) criteria. Olaparib 300 mg orally twice daily was given.

**Results:**

A total of 15 evaluable patients were enrolled. Histology was astrocytoma (*N* = 12) and oligodendroglioma (*N* = 3). Most toxicities were grade 1 or 2. Best response was stable disease (SD) in 9 (60%) patients. Median progression-free survival (PFS) was 3.63 months and median overall survival was 20.7 months. For patients with SD, median PFS was 5.53 months; 4 patients had SD for >6 months. Among patients with best response progressive disease (*N* = 6), 5 had grade 4 tumor and 4 had known *CDKN2A* alteration. PFS was 5.23 months for grades 2 or 3 tumors (*N* = 10) versus 1.8 months for grade 4 (*N* = 5; *P* = 0.0013).

**Conclusion:**

The study did not meet the prespecified response-based activity threshold for moving to step 2. However, prolonged SD was observed in patients with grades 2 and 3 histologies, suggesting olaparib monotherapy could be of clinical benefit in select populations. Grade 4 tumors per 2021 World Health Organization classification defined by histology or *CDKN2A* alteration derived no benefit from this drug, highlighting the usefulness of this classification for future patient stratification and trial design.

**Significance:**

A single-arm phase II trial of olaparib in *IDH*-mutant glioma demonstrated clinically significant prolonged SD for select patients with grade 2/3 disease, suggesting potential benefit of olaparib in *IDH*-mutant gliomas.

## Introduction

Gliomas represent a heterogenous group of diseases that account for most primary brain tumors (1). The discovery of isocitrate dehydrogenase *(IDH*) *1* and *IDH2* mutations (*IDH1/2*mt) in a subset of adult-type diffuse gliomas (2) and the observed improved prognosis associated with this phenotype (3) have led to the incorporation of this disease-defining molecular feature into the World Health Organization (WHO) classification of brain tumors (4). *IDH1*mts occur in more than 70% of what was formerly termed “low-grade” gliomas and up to 20% of “higher-grade” tumors; *IDH2* mutations have been found in about 4% of gliomas (2). Bai and colleagues demonstrated that in gliomas, the *IDH1*mts initially present at diagnosis persist if the tumor recurs (5). The 2021 WHO classification of brain tumors now divides *IDH*mt tumors into oligodendrogliomas, which harbor a 1p/19q codeletion, and astrocytomas, typically characterized by *ATRX* mutations (6). On the basis of histologic features and expected prognosis, oligodendrogliomas can be further graded into WHO grades 2 and 3, and astrocytomas into WHO grades 2 to 4. *IDH*mt astrocytomas with *CDKN2A/B* homozygous deletion are graded as 4 regardless of histology. *IDH*mt gliomas with grade 4 histologic features are no longer termed glioblastoma, given the distinct biology and prognosis.

In general, first-line treatment for gliomas includes maximal safe resection which is often limited by tumor extent and location, adjuvant radiation, and alkylator-based chemotherapy. Chemotherapy options include temozolomide or combination of procarbazine, lomustine, and vincristine (7–11). While a better prognosis is observed in oligodendrogliomas, the disease invariably recurs. Hence novel therapy options are clearly needed.

In addition to its prognostic significance, *IDH1/2*mt is also a promising therapeutic target in the treatment of glioma. The normal function of *IDH* is to catalyze conversion of isocitrate to alpha-ketoglutarate in the citric acid cycle (12). *IDH1/2*mts induce aberrant formation of the oncometabolite 2-hydroxyglutarate (2HG), which is implicated in cancer progression through its inhibitory effect on alpha-ketoglutarate–dependent dioxygenases (13, 14). *IDH* has been successfully targeted in other malignancies that carry *IDH*mt. Currently, two drugs have been approved by the FDA: ivosidenib, approved for *IDH1*mt acute myeloid leukemia (AML) and cholangiocarcinoma, and enasidenib, approved in *IDH2*mt AML (15–17). Trials investigating ivosidenib and vorasidenib in *IDH*mt gliomas have been published recently (18, 19) and are also ongoing (20, 21).

Preclinically, *IDH1/2*mts induce a homologous recombination defect which renders tumor cells sensitive to PARP inhibitors (PARPi; ref. 22). This “BRCAness” phenotype, with sensitivity to PARPi, is due to a single alpha-ketoglutarate–dependent dioxygenase targeted by 2HG, KDM4A, which mediates homologous recombination (22). *IDH1*-dependent PARPi sensitivity has been shown in culture in patient-derived glioma cells as well as genetically matched tumor xenografts *in vivo* (22). This “BRCAness” was significant in *IDH1/2*mt cells and approached a 50-fold difference compared with *IDH1* wildtype cells when exposed to olaparib (22). Olaparib, an orally bioavailable PARPi, has central nervous system (CNS) penetrance (23, 24) and is currently FDA approved for the treatment of several BRCA-mutated tumors (25). We report the results of a multicenter prospective phase II study investigating the role of olaparib monotherapy in recurrent or progressive *IDH1/2*mt gliomas.

## Materials and Methods

### Patient Selection

Eligible patients had recurrent or transformed glioma that progressed despite standard therapy or for which no effective standard therapy existed with evidence of an *IDH1/2*mt associated with neomorphic activity of encoded proteins. On the basis of archived histology, tumors of all grades were eligible, but the presence of tumor contrast enhancement on T1 post-gadolinium MRIs performed prior to enrollment was required for all patients, as a surrogate marker of progression to a high-grade histology. Specific criteria for patient eligibility, tumor classification, and progression (4) can be found in Supplementary Data S1. The representativeness of study participants is discussed in Supplementary Table S1.

### Study Treatment and Design

The primary objective of this trial was to estimate the overall response rate (ORR) of olaparib in patients with *IDH* inhibitor naïve *IDH1/2*mt glioma. Secondary objectives were to estimate the distribution of progression-free survival (PFS), the overall survival (OS) and the duration of response in this population. Safety and tolerability of olaparib were also evaluated.

Treatment cycles were 28 days. Patients were administered olaparib in tablet form orally 300 mg twice daily continually, irrespective of food intake. No premedications were required. Toxicities were graded using the NCI Common Terminology Criteria for Adverse Events version 5.0. Retreatment criteria can be found in Supplementary Data S2. Patients were monitored for disease status using Response Assessment in Neuro-Oncology (RANO) criteria with MRI performed every 8 weeks. Because of preliminary findings suggesting prolonged stable disease (SD) in some patients, an independent review was commissioned. Results were compared with those reported by local investigator. Any discordance was reported and investigated.

Written informed consent was obtained from enrolled patients. Institutional Review Board approvals of the protocol and consent forms were obtained from all sites. Protocol design and conduct complied with all applicable regulations, guidance, and local policies. The study was conducted in accordance with the Declaration of Helsinki. The trial was conducted under an NCI-sponsored Investigational New Drug application (www.ClinicalTrials.gov: NCT03212274). Olaparib was supplied by the Division of Cancer Treatment and Diagnosis of the NCI.

### Statistical Methods

The power analysis and sample size estimation were completed using the Bayesian adaptive trial design method. We modeled the probability of ORR using Beta distributions. The prior distribution is Beta(0.1, 0.9), which is fairly pessimistic; the expected value is 0.1, reflecting the response rate based on previous experiences. We estimated the ORR using the posterior distribution and reported its median and 90% credible interval (see protocol for details). It was predetermined that the trial would be terminated if there was a lack of response, that is, the probability of the ORR being greater than 0.1 was 50% or less. Because the ORR in the trial was zero, the trial was terminated. The SD rate and the progressive disease (PD) rate were reported, and the 90% credible intervals were estimated using the posterior distribution. SD was defined as lack of progression at first restaging after two cycles.

For secondary objectives, the median PFS and OS were estimated using Kaplan–Meier estimate with 95% confidence intervals (CI). The CIs were based on Greenwood formula for variance. Possible risk factors for PFS and OS of WHO grade and *CDKN2A* alteration were compared using the log-rank test. For the secondary objectives, all tests were two sided. Statistical analyses were performed using the R 4.1.1 software.

### Data Availability

The data generated in this study are available upon request from the corresponding author.

## Results

### Patient Demographics

**FIGURE 1:**
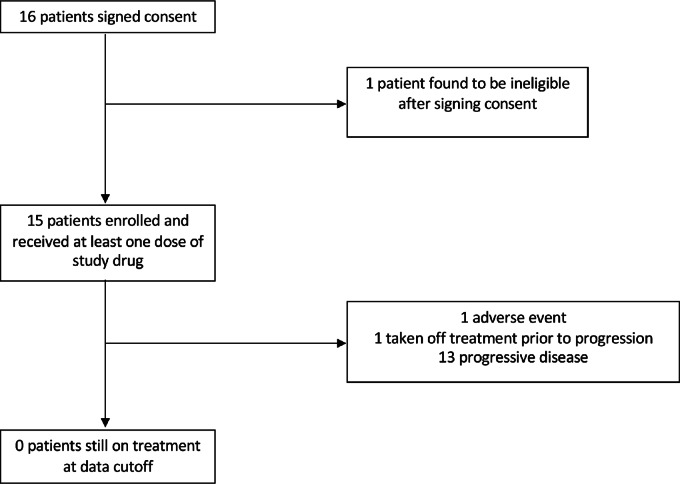
Consort diagram.

Sixteen patients were enrolled. Fifteen patients were evaluable for response and toxicity. One patient was found to be ineligible after signing consent (Fig. 1). Gliomas were further divided according to WHO 2021 grading system by the following subtypes: grades 2 and 3 astrocytomas (n = 7), grade 4 astrocytoma, formerly known as gliblastoma (n = 5), and grades 2 and 3 oligodendrogliomas (n = 3) (Table 1). *IDH*mt was confirmed for each patient, either through prior documentation or testing during enrollment period. Tumors from 13 of 15 patients were found to have mutations that led to the R132H neomorphic phenotype in the *IDH1* gene (one tumor also contained a variant mutation of uncertain significance in *IDH2*) and 2 of 15 patients had tumors which harbored neomorphic mutations in the *IDH2* gene (R172G and R172K; Table 2). *CDKN2A* copy-number loss was documented in 5 patients (Table 2). Patient demographics and prior treatments are summarized in Table 1. All patients had evidence of progression at enrollment.

**TABLE 1:** Demographics and baseline characteristics (*n* = 15)

**Demographics**	
Gender Male Female	10 5
Median (range) age in years	38 (23–69)
Race Caucasian Black	14 1
Performance status 0 1 2	5 9 1
**Original histology**	
Astrocytoma WHO 2016 grade 2 WHO 2016 grade 3	4 5
Glioblastoma WHO 2016 grade 4	3
Oligodendroglioma WHO 2016 grade 2 WHO 2016 grade 3	2 1
IDH mutation status IDH1 IDH2	13 2
**WHO 2021 grading**	
Astrocytoma WHO 2021 grade 2 WHO 2021 grade 3 WHO 2021 grade 4	4 3 5
Oligodendroglioma WHO 2021 grade 2 WHO 2021 grade 3	2 1
**Prior treatment**	
Surgery Surgical resection Biopsy	11 4
Radiation Yes No Median (range) time in years from last radiation to olaparib initiation	14 1 15.48 (0.59–10.59)
**Prior chemotherapy**	
Temozolomide Adjuvant Neoadjuvant	13 10 9
Procarbazine/Vincrinstine/Lomustine Adjuvant Neoadjuvant Bevacizumab Procarbazine/Lomustine Temozolomide/Bevacizumab/Lomustine Carboplatin/Etoposide	3 1 2 2 1 1 1
**Prior immunotherapy**	
Nivolumab	2
**Prior lines of chemotherapy/immunotherapy** 1 2 3 4	5 5 3 2

**TABLE 2:** Summary of patients’ diagnosis and tumor characteristics

Patient	Original histology report	Original histologic grading	Grading by 2021 WHO classification	Date of diagnosis	Surgery	Radiotherapy	Adjuvant therapy	Number of prior treatments	IDH mutation	CDKN2A alteration	ATRX mutation	MGMT promotor methylation	Other alterations/Comments
1	Anaplastic Astrocytoma	3	3	5/2010	Y	Y	Y	3	IDH1 R132H	ND	E1522*	Y	mTP53; TMB low; MS stable; PD-L1<1%; EGFR amplification-ND
2	Anaplastic Astrocytoma	3	4	1/2011	Y	Y	Y	3	IDH1 R132H	CNL	p.K329lfs*3	Y	mTP53; CIC-CNL
3	Low grade astrocytoma/glioma	2	2	12/2014	Biopsy only	Y	Y	3	IDH2 R172G	ND	Q468fs*4	NR	mTP53; mCHEK2; TMB low; MS stable
4	Infiltrating glioma with astrocytic phenotype	3	4	4/2015	Y	Y	Y	1	IDH1 R132H	CNL	LON^i^	Y	1 q status conserved; 19 q status conserved; mTP53; PTEN-CNL Low level amplification of MYC and MYCN
5	Anaplastic Astrocytoma	3	3	3/2012	Biopsy only	Y	Y	4	IDH1 R132H	ND	NR	Y	mTP53; EGFR amplification-ND 1 q status conserved; 19 q status conserved; PTEN-33% loss; Chromosome 17p-100% loss
6	Infiltrating Astrocytoma	2	2	9/2012	Biopsy only	Y	N	1	IDH1 R132H	ND	NR	Y	1 q status conserved; 19 q status conserved; p53 positive; EGFR amplification-ND
7	Anaplastic oligodendroglioma	3	3	11/2005	Biopsy only	Y	Y	4	IDH1 R132H IDH2 VUS	ND	VUS-pT578i	Y	1p 9q deletion; TP53 deletion TERT promoter mutation; EGFR positive (no amplification) CIC-CNL FUBP1-CNL VUS-PTCH1, NF2, PI3K
8	Diffuse astrocytoma	2	2	7/2012	Y	N	Y	2	IDH1 R132H	ND	NR	Y	EGFR positive (no amplification); 1 p, 19q, p16, PTEN all conserved; TP53 deletion positive
9	Glioblastoma	4	4	12/2016	Y	Y	Y	1	IDH1 R132H	CNL	Y	N	EGFR-not amplified; 1p and 19q conserved
10	Diffuse astrocytoma	2	2	7/2014	Y	Y	Y	1	IDH1 R132H	ND	pR808* (LOF)	Y	mTP53; TMB low; MS stable; PDL-1 low
11	Glioblastoma	4	4	6/2008	Y	Y	Y	1	IDH1 R132H	CNL	Y	N	GFAP positive; TP53 positive EGFR positive MET amplification
12	Anaplastic Astrocytoma	3	3	3/2010	Y	Y	Y	2	IDH2 R172K	NR	NR	N	Polysomies of both chromosome 1 and 19, no losses; No mutation of TERT promoter
13	Oligodendroglioma	2	2	2/2008	Y	Y	Y	2	IDH1 R132H	NR	NR	NR	mPI3CA; mTERT promoter; TMB low; MS stable; PDL-1 low
14	Oligodendroglioma	2	2	12/2008	Y	Y	Y	2	IDH1 R132H	NR	NR	NR	TMB low; MS stable; mTP53;
15	Glioblastoma	4	4	5/2018	Y	Y	Y	2	IDH1 R132H	CNL	p.Q8883Rfs*21 and LON^i^	N	mTP53; PtEN-CNL 1 p and 19 q abnormalities

Abbreviations: CNL, copy-number loss; LOF, loss of function; MS, microsatellite stability; N = No; ND, no genetic alteration detected; NR, not reported; TMB, tumor mutational burden; VUS, variant of unknown significance; Y, Yes.

### Toxicity

There were no unexpected toxicities reported and most toxicities were grade 1 or 2. The most common toxicities were nausea (67%) and fatigue (47%; Table 3). One patient with a history of ulcerative colitis reported grade 3 diarrhea that resulted in a dose reduction to 250 mg twice daily after the second cycle. The patient was taken off study during cycle 5 due to continued drug-related diarrhea. One patient developed grade 3 hypertension that was possibly attributed to olaparib. Onset was after patient stopped treatment at cycle 12 and subsequently improved at follow-up visit. Finally, grade 3 lymphopenia was reported in 1 patient.

**TABLE 3:** Most common (>10%) treatment-related and grade 1–3 treatment-related adverse events (maximum grade, all cycles; *n* = 15). No grade 4 or 5 events were reported

Adverse event	Total number of patients with event *N* (%)	Grade 1 (%)	Grade 2 (%)	Grade 3 (%)
Nausea	10 (67%)	9 (90%)	1 (10%)	—
Fatigue	7 (47%)	4 (57%)	3 (43%)	—
Anemia	4 (27%)	4 (100%)	—	—
Elevated LDH	4 (27%)	4 (100%)	—	—
Vomiting	4 (27%)	4 (100%)	—	—
Diarrhea	4 (27%)	2 (50%)	1 (25%)	1 (25%)
Lymphopenia	3 (20%)	2 (67%)	—	1 (33%)
Thrombocytopenia	3 (20%)	3 (100%)	—	—
Anorexia	2 (13%)	2 (100%)	—	—
Leukopenia	2 (13%)	2 (100%)	—	—
Neutropenia	2 (13%)	2 (100%)	—	—
Hypertension	1 (7%)	—	—	1 (100%)

### Efficacy

All 15 patients were evaluable for response (Table 4). Nine (60%, 90% credible interval: 37–76) patients achieved SD and 6 (40%, 90% credible interval: 19–58) patients progressed. For patients with SD, the median number of progression-free months was 5.53 (range, 2.83–12.13). Four patients had SD for > 6 months (Table 4). The remaining 6 patients experienced PD after two cycles with a median of 1.82 months on treatment (range, 1.8–1.9).

**TABLE 4:** Patients’ response to olaparib

Patient	Original histology report	2021 WHO classification	Overall best response	Number of cycles completed	Days on treatment	Months on treatment	Progression-free survival (months)	Overall survival (months)
1	Anaplastic Astrocytoma	3	SD	10^a^	278^a^	9.3	5.5	10.9+
2	Anaplastic Astrocytoma	4	PD	2	56	1.9	1.8	9.1
3	Low-grade astrocytoma/glioma	2	SD	12	339	11.3	11.3	12.5+
4	Infiltrating glioma with astrocytic phenotype	4	PD	2	54	1.8	1.8	13.8
5	Anaplastic Astrocytoma	3	PD	2	56	1.9	1.9	32.1+
6	Infiltrating Astrocytoma	2	PD	2	55	1.8	1.8	8.3
7	Anaplastic oligodendroglioma	3	SD	5	149	5	4.9	31.5+
8	Diffuse astrocytoma	2	SD	8	224	7.5	7.4	20.7
9	Glioblastoma	4	SD	4	105	3.5	2.8	16.6
10	Diffuse astrocytoma	2	SD	14	364	12.1	12.1	29.5
11	Glioblastoma	4	PD	2	57	1.9	1.9	3.2+
12	Anaplastic Astrocytoma	3	SD	10	279	9.3	9.2	10.9+
13	Oligodendroglioma	2	SD	4	110	3.7	3.6	19.9
14	Oligodendroglioma	2	SD	5	120	4.0	4	9.1+
15	Glioblastoma	4	PD	2	54	1.8	1.8	26.1+

NOTE: PFS defined as the duration of time from start of treatment to time of progression or death, whichever occurs first. OS calculated from initiation of treatment to date of death or last known date of contact. + = alive or lost to follow-up.

^a^Results of review of patient's MRIs by CTEP-appointed independent neuroradiologist demonstrated progressive disease at end of cycle 6 (day 166 of study). Patient was treated for four additional cycles after progression.

**FIGURE 2:**
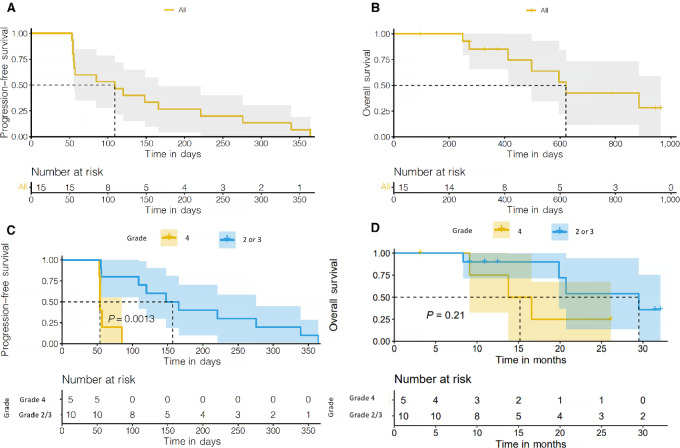
Kaplan–Meier survival curves. **A,** PFS among all patients. **B,** OS among all patients. **C,** PFS comparing patients with grade 4 disease by histology or CDKN2A alteration to those with grade 2 or 3 disease (the log-rank test *P* value is reported). **D,** OS comparing patients with grade 4 disease by histology or CDKN2A alteration to those with grades 2 or 3 disease (log-rank test *P* value is reported).

In the whole study population, the median PFS was 3.63 months (95% CI, 1.87–5.53; Fig. 2A), median OS was 20.73 months (95% CI, 13.77–not reached [NR]; Fig. 2B), median number of cycles completed was 4 (range, 2–14), and the median number of months on treatment was 3.7 (range, 1.8–12.1). The study did not meet the prespecified response-based threshold for moving to stage 2.

We compared PFS and OS of patients with WHO grade 2 or 3 lesions based on archived tissue from diagnosis, to those with grade 4 by histology or CDKN2A alteration. The median PFS of patients with grade 2 and/or 3 was 5.23 months (95% CI, 3.63–9.2) while those with grade 4 was 1.8 months (95% CI, 1.77–NR; log-rank test *P* = 0.0013 Fig. 2C). The median OS of patients with grade 2/3 disease was 29.5 months (95% CI, 19.87–NR) vs. 15.17 months (95% CI, 9.1–NR) for grade 4 tumors (*P* = 0.21; Fig. 2D).

Reevaluation of imaging of all patients by a blinded NCI-designated neuroradiologist was performed. Two discordant results were identified: one patient's tumor had progressed after six cycles of treatment but was kept on for four additional cycles by the enrolling site. The corrected months of progression-free disease was reported in the above data. The second patient with discordant results had been taken off treatment due to PD after five cycles by their treating physician. However, reassessment of their scans demonstrated SD.

## Discussion

Olaparib monotherapy was well tolerated in patients with recurrent or progressive contrast-enhancing *IDH1/2*mt glioma but did not meet prespecified response-based activity criteria for moving to stage 2 of the trial. However, clinically significant prolonged SD was observed in a relatively large proportion of patients, particularly those with WHO grades 2 and 3 histologies, suggesting olaparib monotherapy could be of clinical benefit in select patients. Grade 4 tumors, as defined by the 2021 WHO classification using histology or presence of *CDKN2A* deletion, derived minimal or no benefit from this drug demonstrating the utility of this new classification for possible future patient stratification and trial design.

PARP is important in the repair of single-strand breaks (26). In PARP inhibited cells, single-strand breaks accumulate and become more lethal double-strand breaks in the process of DNA replication. Cells with BRCA1 or BRCA2 mutations have defects in homologous recombination repair of double-strand DNA breaks. Patients with BRCA1- and BRCA2-mutated tumors treated with PARPis often have dramatic treatment responses as the synthetic lethality induced by the PARPi leads to cell death within the tumor (27, 28). Favorable responses to PARPi have also been seen in tumors with DNA repair alterations other than BRCA mutations (29, 30). Despite preclinical susceptibility of *IDH1/2*mt tumors to PARPi, our trial did not meet the prespecified endpoint of ORR to unequivocally demonstrate biological activity deriving from the “BRCAness” effects associated with 2-HG. However, the PFS data and disease stability observed in a relatively large proportion of patients (60%) raises the question of whether PARPi could still be of clinical benefit in these patients, in the absence of measurable tumor shrinkage. While our findings may suggest potential benefit in patients with lower grade histologies or at earlier stages of disease progression, further investigation is required as such phenotypes are inherently less aggressive. Other trials have studied PARPi in patients with gliomas, but their inclusion criteria likely excluded most patients with *IDH1/2*mt and/or other homologous recombination defects (31, 32). In our trial, selection of patients based on contrast enhancement was an attempt to focus on the patients with *IDH*mt tumors of a higher grade or more aggressive behavior. However, the molecular heterogeneity of patients included in our study, patients’ enrollment at varying stages in their tumor's natural history, the small number of patients with oligodendroglioma, and differences in timing of collection of molecular information (at diagnosis vs. at time of recurrence) limit the conclusions that can be drawn from our data. Regardless, it is clear from the results of this trial that single-agent olaparib is not a sufficient treatment for grade 4 gliomas.

Recently, preliminary results of the OLAGLI trial investigating olaparib in 35 patients with recurrent *IDH1/2*mt high-grade contrast-enhancing gliomas were presented at American Society of Clinical Oncology 2021 (33). That study found a 6-month PFS rate of 31%, which did not meet prespecified threshold for significance, but 2 patients (5%) had a PR and 14 patients (40%) achieved SD. The median duration of response in the combined PR and SD patients was 9 months. The median OS was 15.9 months (33). Similar to our study, these preliminary results suggest that there may be patients who experience clinically meaningful benefit from single-agent PARPi through disease stability, although olaparib's activity as a single agent as measured by radiographic responses and tumor shrinkage is modest.

An important concern in the development of novel therapies for CNS tumors is the ability of novel agents to penetrate through areas of intact blood–brain barrier to address infiltrative disease. CNS penetration of olaparib in humans has been shown in a phase I study combining temozolomide with olaparib in patients with relapsed glioblastoma; olaparib, given prior to surgery, was detected in 71 of 71 tumor core specimens from 27 patients (34). Detected drug levels were greater than 100 nmol/L, the concentration determined by *in vitro* experiments to be needed for maximal PARP inhibition (35). Trials are ongoing investigating novel PARPis with potential for even greater brain penetration (NCT00687765, NCT03914742).

Identifying mutations that further define distinct subgroups within *IDH*mt glioma for updated grading and selection of patients who may benefit from PARPi remains of high interest. Homozygous deletion of *CDKN2A* has been noted to be an adverse prognostic factor associated with worse PFS and OS in patients with *IDH1/2*mt disease (36, 37) and per the 2021 WHO classification of tumors confers grade 4 status (6). In our study, 4 of 5 patients with known *CDKN2A* alteration progressed on first restaging. Our findings therefore seem to support the inclusion of *CDKN2A* status as a grade-defining molecular finding for the grading of gliomas, as acknowledged in this new classification of gliomas by the WHO. A limitation in our observation is that *CDKN2A* copy-number status was assessed by next-generation sequencing (NGS) performed locally, and the distinction between homozygous and heterozygous deletion was not uniformly provided. While in *IDH* wildtype glioblastoma *CDKN2A* deletions are nearly universally homozygous, we acknowledge that in some patients with *IDH1/2*mt gliomas, the *CDKN2A* deletion as determined by NGS may not be homozygous. Therefore, it is possible, but unlikely, that the 2 patients with poor outcomes that were reclassified as grade 4 per WHO 2021 may not meet criteria for such, should other methods for *CDKN2A* evaluation be utilized.

In addition to PARPi, a significant focus of research in *IDH1/2*mt gliomas is on direct inhibition of *IDH*mt proteins. In a phase I trial, 30 patients with recurrent or progressive *IDH1/2*mt enhancing glioma received vorasidenib, an oral, brain-penetrant inhibitor of *IDH1/2*mt. ORR was 0% but 56.7% of patients achieved SD. The median PFS was 3.6 months, similar to our trial (19). A window-of-opportunity trial of vorasidenib and the *IDH1* inhibitor ivosidenib randomized patients with recurrent nonenhancing WHO 2016 grade 2 or 3 *IDH1*mt glioma who were undergoing craniotomy to receive either ivosidenib, vorasidenib, or no treatment for 4 weeks preoperatively. Postoperatively, they were randomized to ivosidenib or vorasidenib. Both drugs were found to be CNS penetrant (brain:plasma ratio 0.16 for ivosidenib and 2.4 for vorasidenib) and both lowered 2HG levels compared with untreated samples (21). ORR was 30.8%. Switching to a focus on earlier stage disease, the phase III INDIGO trial is randomizing *IDH1/2*mt residual or recurrent grade 2, nonenhancing glioma to receive vorasidenib or placebo, with primary endpoint of PFS (38).

There are numerous future study directions in this population. Trials investigating timing of *IDH*-targeted therapy with *IDH* inhibitors or PARPi in the first-line (NCT03581292) or recurrent/relapsed setting and timing with respect to surgery will be important. An ongoing study is investigating the effect of niraparib prior to surgery in *IDH*mt glioma (NCT05076513). Studies that compare responses in patients with contrast-enhancing and non–contrast-enhancing tumors will also provide information on which populations are most likely to benefit. Outcomes of treatment with PARPi in patients who have received prior treatment with IDH inhibitors are currently being investigated in a phase II clinical trial (NCT03212274). Finally, it is possible that PARPi may require combination with additional therapies to improve activity and induce tumor shrinkage. A single-center retrospective study reported the results of patients with glioma, some with *IDH1/*2mt, who were treated with combination olaparib and temozolomide. Four of the 8 patients with grade 2 or 3 *IDH*mt disease had complete or partial response. Consistent with our findings, no responses were seen in patients with grade 4 *IDH*mt glioma. This trial suggests promise of olaparib in combination but is limited by its retrospective design (39). Other ongoing trials investigating combination therapy include PARPi + PD-L1 inhibitor (NCT03991832), PARPi + temozolomide (NCT02152982, NCT04552977, NCT01390571, NCT04910022, NCT03581292, NCT03749187, NCT03914742, NCT03212742), and PARPi + temozolomide + radiotherapy (NCT04614909). Taken together, this body of work will establish whether PARPi will eventually play a meaningful role in the treatment of gliomas.

## Supplementary Material

Supplementary Data Definition of Progression, Tumor Classification, and Eligibility Criteria, retreatment criteria

Supplementary Table 1 Representativeness of Study Participants

## References

[1] Ostrom QT , Gittleman H, Xu J, Kromer C, Wolinsky Y, Kruchko C, . CBTRUS statistical report: primary brain and other central nervous system tumors diagnosed in the United States in 2009–2013. Neuro Oncol 2016;18:v1–75. 28475809 10.1093/neuonc/now207 PMC8483569

[2] Yan H , Parsons DW, Jin G, McLendon R, Rasheed BA, Yuan W, . IDH1 and IDH2 mutations in gliomas. N Engl J Med 2009;360:765–73. 19228619 10.1056/NEJMoa0808710 PMC2820383

[3] Reuss DE , Mamatjan Y, Schrimpf D, Capper D, Hovestadt V, Kratz A, . IDH mutant diffuse and anaplastic astrocytomas have similar age at presentation and little difference in survival: a grading problem for WHO. Acta Neuropathol 2015;129:867–73. 25962792 10.1007/s00401-015-1438-8 PMC4500039

[4] Louis DN , Perry A, Reifenberger G, von Deimling A, Figarella-Branger D, Cavenee WK, . The 2016 World Health Organization classification of tumors of the central nervous system: a summary. Acta Neuropathol 2016;131:803–20. 27157931 10.1007/s00401-016-1545-1

[5] Bai H , Harmancı AS, Erson-Omay EZ, Li J, Coşkun S, Simon M, . Integrated genomic characterization of IDH1-mutant glioma malignant progression. Nat Genet 2016;48:59–66. 26618343 10.1038/ng.3457 PMC4829945

[6] Louis DN , Perry A, Wesseling P, Brat DJ, Cree IA, Figarella-Branger D, . The 2021 WHO classification of tumors of the central nervous system: a summary. Neuro-oncol 2021;23:1231–51. 34185076 10.1093/neuonc/noab106 PMC8328013

[7] Bell EH , Zhang P, Shaw EG, Buckner JC, Barger GR, Bullard DE, . Comprehensive genomic analysis in NRG oncology/RTOG 9802: a phase III trial of radiation versus radiation plus procarbazine, lomustine (CCNU), and vincristine in high-risk low-grade glioma. J Clin Oncol 2020;38:3407–17. 32706640 10.1200/JCO.19.02983 PMC7527157

[8] van den Bent MJ , Brandes AA, Taphoorn MJ, Kros JM, Kouwenhoven MC, Delattre JY, . Adjuvant procarbazine, lomustine, and vincristine chemotherapy in newly diagnosed anaplastic oligodendroglioma: long-term follow-up of EORTC brain tumor group study 26951. J Clin Oncol 2013;31:344–50. 23071237 10.1200/JCO.2012.43.2229

[9] van den Bent MJ , Tesileanu CMS, Wick W, Sanson M, Brandes AA, Clement PM, . Adjuvant and concurrent temozolomide for 1p/19q non-co-deleted anaplastic glioma (CATNON; EORTC study 26053–22054): second interim analysis of a randomised, open-label, phase 3 study. Lancet Oncol 2021;22:813–23. 34000245 10.1016/S1470-2045(21)00090-5 PMC8191233

[10] Fisher BJ , Hu C, Macdonald DR, Lesser GJ, Coons SW, Brachman DG, . Phase 2 study of temozolomide-based chemoradiation therapy for high-risk low-grade gliomas: preliminary results of radiation therapy oncology group 0424. Int J Radiat Oncol Biol Phys 2015;91:497–504. 25680596 10.1016/j.ijrobp.2014.11.012 PMC4329190

[11] Gilbert MR , Wang M, Aldape KD, Stupp R, Hegi ME, Jaeckle KA, . Dose-dense temozolomide for newly diagnosed glioblastoma: a randomized phase III clinical trial. J Clin Oncol 2013;31:4085–91. 24101040 10.1200/JCO.2013.49.6968 PMC3816958

[12] Han S , Liu Y, Cai SJ, Qian M, Ding J, Larion M, . IDH mutation in glioma: molecular mechanisms and potential therapeutic targets. Br J Cancer 2020;122:1580–9. 32291392 10.1038/s41416-020-0814-x PMC7250901

[13] Dang L , White DW, Gross S, Bennett BD, Bittinger MA, Driggers EM, . Cancer-associated IDH1 mutations produce 2-hydroxyglutarate. Nature 2010;465:966. 20559394 10.1038/nature09132 PMC3766976

[14] Xu W , Yang H, Liu Y, Yang Y, Wang P, Kim SH, . Oncometabolite 2-hydroxyglutarate is a competitive inhibitor of α-ketoglutarate-dependent dioxygenases. Cancer Cell 2011;19:17–30. 21251613 10.1016/j.ccr.2010.12.014 PMC3229304

[15] Stein EM , Fathi AT, DiNardo CD, Pollyea DA, Roboz GJ, Collins R, . Enasidenib in patients with mutant IDH2 myelodysplastic syndromes: a phase 1 subgroup analysis of the multicentre, AG221-C-001 trial. Lancet Haematol 2020;7:e309–19. 32145771 10.1016/S2352-3026(19)30284-4

[16] DiNardo CD , Stein EM, de Botton S, Roboz GJ, Altman JK, Mims AS, . Durable remissions with ivosidenib in IDH1-mutated relapsed or refractory AML. N Engl J Med 2018;378:2386–98. 29860938 10.1056/NEJMoa1716984

[17] Abou-Alfa GK , Macarulla T, Javle MM, Kelley RK, Lubner SJ, Adeva J, . Ivosidenib in IDH1-mutant, chemotherapy-refractory cholangiocarcinoma (ClarIDHy): a multicentre, randomised, double-blind, placebo-controlled, phase 3 study. Lancet Oncol 2020;21:796–807. 32416072 10.1016/S1470-2045(20)30157-1 PMC7523268

[18] Mellinghoff IK , Ellingson BM, Touat M, Maher E, De La Fuente MI, Holdhoff M, . Ivosidenib in isocitrate dehydrogenase 1-mutated advanced glioma. J Clin Oncol 2020;38:3398–406. 32530764 10.1200/JCO.19.03327 PMC7527160

[19] Mellinghoff IK , Penas-Prado M, Peters KB, Burris HA 3rd, Maher EA, Janku F, . Vorasidenib, a dual inhibitor of mutant IDH1/2, in recurrent or progressive glioma; results of a first-in-human phase I trial. Clin Cancer Res 2021;27:4491–9. 34078652 10.1158/1078-0432.CCR-21-0611 PMC8364866

[20] Mellinghoff IK , Peters KB, Cloughesy TF, Burris Iii HA, Maher EA, Janku F, . Vorasidenib (VOR; AG-881), an inhibitor of mutant IDH1 and IDH2, in patients (pts) with recurrent/progressive glioma: updated results from the phase I non-enhancing glioma population. J Clin Oncol 2020;38:15s, (suppl; abstr 2504).

[21] Mellinghoff IK , Wen PY, Taylor JW, Maher EA, Arrillaga-Romany I, Peters KB, . PL3.1 A phase 1, open-label, perioperative study of ivosidenib (AG-120) and vorasidenib (AG-881) in recurrent, IDH1-mutant, low-grade glioma: results from cohort 1. Neuro Oncol 2019;21:iii2.

[22] Sulkowski PL , Corso CD, Robinson ND, Scanlon SE, Purshouse KR, Bai H, . 2-Hydroxyglutarate produced by neomorphic IDH mutations suppresses homologous recombination and induces PARP inhibitor sensitivity. Sci Transl Med 2017;9:eaal2463. 28148839 10.1126/scitranslmed.aal2463 PMC5435119

[23] Salinas B , Irwin CP, Kossatz S, Bolaender A, Chiosis G, Pillarsetty N, . Radioiodinated PARP1 tracers for glioblastoma imaging. EJNMMI Res 2015;5:123. 26337803 10.1186/s13550-015-0123-1 PMC4559561

[24] Lesueur P , Lequesne J, Grellard JM, Dugué A, Coquan E, Brachet PE, . Phase I/IIa study of concomitant radiotherapy with olaparib and temozolomide in unresectable or partially resectable glioblastoma: OLA-TMZ-RTE-01 trial protocol. BMC Cancer 2019;19:198. 30832617 10.1186/s12885-019-5413-y PMC6399862

[25] Bochum S , Berger S, Martens UM. Recent Results Cancer Res 2018;211:217–33. 30069770 10.1007/978-3-319-91442-8_15

[26] Murai J , Huang SY, Renaud A, Zhang Y, Ji J, Takeda S, . Stereospecific PARP trapping by BMN 673 and comparison with olaparib and rucaparib. Mol Cancer Ther 2014;13:433–43. 24356813 10.1158/1535-7163.MCT-13-0803 PMC3946062

[27] Zhao W , Wiese C, Kwon Y, Hromas R, Sung P. The BRCA tumor suppressor network in chromosome damage repair by homologous recombination. Annu Rev Biochem 2019;88:221–45. 30917004 10.1146/annurev-biochem-013118-111058 PMC7004434

[28] Lord CJ , Ashworth A. PARP inhibitors: synthetic lethality in the clinic. Science 2017;355:1152–8. 28302823 10.1126/science.aam7344 PMC6175050

[29] Bryant HE , Schultz N, Thomas HD, Parker KM, Flower D, Lopez E, . Specific killing of BRCA2-deficient tumours with inhibitors of poly(ADP-ribose) polymerase. Nature 2005;434:913–7. 15829966 10.1038/nature03443

[30] Farmer H , McCabe N, Lord CJ, Tutt AN, Johnson DA, Richardson TB, . Targeting the DNA repair defect in BRCA mutant cells as a therapeutic strategy. Nature 2005;434:917–21. 15829967 10.1038/nature03445

[31] Chalmers AJ , Jackson A, Swaisland H, Stewart W, Halford SE, Molife LR, . Results of stage 1 of the oparatic trial: a phase I study of olaparib in combination with temozolomide in patients with relapsed glioblastoma. J Clin Oncol 2014;32:15s,(suppl; abstr 2025).

[32] Myung JK , Cho HJ, Kim H, Park CK, Lee SH, Choi SH, . Prognosis of glioblastoma with oligodendroglioma component is associated with the IDH1 mutation and MGMT methylation status. Transl Oncol 2014;7:712–9. 25500080 10.1016/j.tranon.2014.10.002 PMC4311043

[33] Ducray F , Sanson M, Chinot OL, Fontanilles M, Rivoirard R, Thomas-Maisonneuve L, . Olaparib in recurrent IDH-mutant high-grade glioma (OLAGLI). J Clin Oncol 2021;39:15s, (suppl; abstr 2007).

[34] Hanna C , Kurian KM, Williams K, Watts C, Jackson A, Carruthers R, . Pharmacokinetics, safety, and tolerability of olaparib and temozolomide for recurrent glioblastoma: results of the phase I OPARATIC trial. Neuro Oncol 2020;22:1840–50. 32347934 10.1093/neuonc/noaa104 PMC7746945

[35] 206162 Clinical Pharmacology Review; 2014.

[36] Appay R , Dehais C, Maurage CA, Alentorn A, Carpentier C, Colin C, . CDKN2A homozygous deletion is a strong adverse prognosis factor in diffuse malignant IDH-mutant gliomas. Neuro Oncol 2019;21:1519–28. 31832685 10.1093/neuonc/noz124 PMC7145561

[37] Shirahata M , Ono T, Stichel D, Schrimpf D, Reuss DE, Sahm F, . Novel, improved grading system(s) for IDH-mutant astrocytic gliomas. Acta Neuropathol 2018;136:153–66. 29687258 10.1007/s00401-018-1849-4

[38] Mellinghoff I , van den Bent M, Clarke J, Maher E, Peters K, Touat M, . RTID-05. Indigo: a global, randomized, double-blind, Phase 3 study of vorasidenib (AG-881) vs placebo in patients with residual/recurrent grade II glioma with an isocitrate dehydrogenase 1/2 (IDH1/2) mutation. Neuro Oncol 2020;22:ii194.

[39] Schaff LR , Kushnirsky M, Lin AL, Nandakumar S, Grommes C, Miller AM, . Combination olaparib and temozolomide for the treatment of glioma: a retrospective case series. Neurology 2022;99:750–5. 35948444 10.1212/WNL.0000000000201203 PMC9620814

