# Supplementary material for: Multicenter Phase II Trial of the PARP Inhibitor Olaparib in Recurrent IDH1- and IDH2-mutant Glioma
Source: Cancer Res Commun. 2023 Feb 2;3(2):192–201. doi: 10.1158/2767-9764.CRC-22-0436 (PMC10035510; doi:10.1158/2767-9764.CRC-22-0436)
Supplement: Supplementary Data — Definition of Progression, Tumor Classification, and Eligibility Criteria, retreatment criteria [file crc-22-0436-s01.docx]

**Supplement 1: Definition of Progression, Tumor Classification, and Eligibility Criteria**

**Patients with WHO Grade III or IV Glioma and progressive disease < 12 weeks
after completion of chemoradiotherapy**

- New enhancement outside of the radiation field (beyond the high-dose regions or 80% isodose line)
- Unequivocal evidence of viable tumor on histopathologic sampling
  (e.g., solid tumor areas. i.e., >70% tumor cell nuclei in areas), high or
  progressive increase in MIB-1 proliferation index compared with prior biopsy,
  or evidence for histologic progression or increased anaplasia in tumor).
- Note: Given the difficulty of differentiating true progression from
  pseudoprogression, clinical decline alone, in the absence of radiographic or
  histologic confirmation of progression, was not sufficient for definition of
  progressive disease in the first 12 weeks after completion of concurrent
  chemoradiotherapy.

**Patients with WHO Grade III or IV glioma and progressive disease ≥12 weeks after
completion of chemoradiotherapy**

- New contrast-enhancing lesion outside of radiation field on decreasing, stable,
  or increasing doses of corticosteroids.
- Increase by ≥ 25% in the sum of the products of perpendicular diameters
  between the first post-radiotherapy scan, or a subsequent scan with smaller
  tumor size, and the scan at 12 weeks or later on stable or increasing doses of
  corticosteroids.
- For patients who received antiangiogenic therapy, significant increase in
  T2/FLAIR non-enhancing lesion was also considered progressive disease.
  The increased T2/FLAIR must have occurred with the patient on stable or
  increasing doses of corticosteroids compared with baseline scan or best
  response after initiation of therapy and not be a result of comorbid events (e.g.,
  effects of radiation therapy, demyelination, ischemic injury, infection, seizures,
  postoperative changes, or other treatment effects).
- Note: Clinical deterioration alone (not attributable to concurrent medication or
  comorbid conditions) was sufficient to declare progression on current treatment but not for entry onto the clinical trial for recurrence.

**Patients with WHO Grade II glioma progression**

- Development of new lesions or increase of enhancement (radiological
  evidence of malignant transformation)
- A 25% increase of the T2 or FLAIR non-enhancing lesion on stable or
  increasing doses of corticosteroids compared with baseline scan or best
  response after initiation of therapy, not attributable to radiation effect or to
  comorbid events

**2021 WHO Classification of CNS Tumors (1)**

Astrocytoma, IDH-mutant

- Genes/molecular profiles characteristically altered: IDH1, IDH2, ATRX, TP53, CDKN2A/B
- Grades 2, 3, 4 based on histology and presence of CDKN2A/B deletion.
- Presence of CDKN2A/B homozygous deletion results in a CNS WHO grade of 4 regardless of microvascular proliferation or necrosis

Oligodendroglioma, IDH-mutant, and 1q/19q-codeleted

- Genes/molecular profiles characteristically altered: IDH1, IDH2, 1p/19q, TERT promoter, CIC, FUBP1, NOTCH1
- Grades 2, 3 based on histology

**Eligibility Criteria**

IDHmts were determined using an FDA-approved molecular test or validated DNA-based assay conducted in a Clinical Laboratory Improvement Amendments (CLIA)-certified laboratory. Only specific mutations that were confirmed to lead to a neomorphic phenotype were eligible for enrollment. Patients were not previously treated with an IDH inhibitor. Patients must have had Eastern Cooperative Oncology Group (ECOG) performance status 0-2; measurable disease (per Response Assessment in Neuro-Oncology (RANO) criteria (2)) and adequate organ and marrow function defined as absolute neutrophil count ≥ 1,500/μL, platelets ≥100,000/μL, aspartate aminotransferase and/or alanine aminotransferase ≤ 2.5 x upper limit of normal (ULN) or ≤ 5 x ULN if liver metastases present; bilirubin ≤ 1.5 x ULN; and creatinine clearance ≥ 51 mL/min/1.73 m^2^ calculated by the Cockcroft-Gault equation.

Prior chemotherapy, surgery and radiotherapy (except for palliative reasons) must have been completed at least 3 weeks prior to treatment initiation. Prior treatment with an investigational agent must have been > 30 days or five half-lives of the drug (whichever was less) prior to initiation of study treatment. Toxicities from these agents must have recovered to < grade 1, with the exception of stable chronic grade 2 toxicities that did not overlap with presumed toxicities of olaparib. Prior PARPi exposure was not permitted. CYP3A substrates had to be discontinued at least 3 weeks prior to the first administration of olaparib. Patients were also excluded if they had an uncontrolled intercurrent illness, known active hepatitis or HIV, or suffered profound neurological symptoms from their disease. The use of stable doses of corticosteroids was allowed if these agents were started at least 4 weeks prior to treatment.

**Supplement 2: Retreatment Criteria**

A new cycle of therapy did not begin until toxicities recovered to grade <1 with no more than a 4-week delay permitted. Exceptions to this stipulation were in the management of anemia, neutropenia, leukopenia, and thrombocytopenia. With these toxicities, based on investigator’s judgment, patients were allowed to continue on olaparib without delay or dose reduction if toxicity grade was <2. In patients experiencing anemia at a grade > 2, supportive care (e.g. transfusion) was allowed. If repeat hemoglobin was < 10 gm/dl but > 8 gm/dl, dosing was interrupted (for a maximum of 4 weeks) until hemoglobin > 10 mg/dl and upon recovery, patient was dosed with a reduction to 250 mg twice daily as initial de-escalation. Patient was allowed a second de-escalation to 200 mg twice daily if such toxicity recurred. For a grade >3 toxicity, treatment was resumed at one dose level reduction (i.e. 250 mg twice daily) once toxicity recovered to <grade 1. Patients who experienced neutropenia, leukopenia or thrombocytopenia at a grade > 3 were allowed to recover to a grade <1 with no dose reduction required on the first occurrence. Patients experiencing a repeat occurrence of neutropenia, leukopenia, or thrombocytopenia at a grade >3, received a dose reduction of olaparib as described above. Dose re-escalation and reductions beyond 200 mg twice daily were not allowed.

1. Louis DN, Perry A, Wesseling P, Brat DJ, Cree IA, Figarella-Branger D, et al. The 2021 WHO classification of tumors of the central nervous system: a summary. Neuro-oncology. 2021;23(8):1231-51.

2. Wen PY, Macdonald DR, Reardon DA, Cloughesy TF, Sorensen AG, Galanis E, et al. Updated response assessment criteria for high-grade gliomas: response assessment in neuro-oncology working group. J Clin Oncol. 2010;28(11):1963-72.
