# Supplementary material for: Multicenter Phase II Trial of the PARP Inhibitor Olaparib in Recurrent IDH1- and IDH2-mutant Glioma
Source: Cancer Res Commun. 2023 Feb 2;3(2):192–201. doi: 10.1158/2767-9764.CRC-22-0436 (PMC10035510; doi:10.1158/2767-9764.CRC-22-0436)
Supplement: Supplementary Table 1 — Representativeness of Study Participants [file crc-22-0436-s02.docx]

**Supplementary table 1:** Representativeness of Study Participants

Cancer Type: Glioma

Considerations related to:

| **Sex** | These tumors are more common in men, with incidence rate ratio of glioblastoma male:female of 1.57, oligodendroglioma 1.34, other astrocytoma (excluding glioblastoma and pilocytic astrocytoma) 1.32.(3) |
| --- | --- |
| **Age** | The median age of diagnosis for tumors of neuophithelial tissue, which includes the gliomas discussed in this study, is 56. Median age of diagnosis of anaplastic astrocytoma is 53 while for glioblastoma it is 64.(3) |
| **Race/ethnicity** | Astrocytomas and oligodendrogliomas are more common in white patients than black patients. Incidence rate ratios (white:black) are 1.93 for glioblastoma, 1.84 for all other astrocytoma (excluding glioblastoma and pilocytic astrocytoma), 2.55 for oligodendroglioma.(3) |
| **Geography** | Patients in the US with primary CNS tumors make up about 8% of diagnoses of this condition worldwide. (4) |
| **Overall representativeness of this study** | The median age of patients in our trial was younger than the population of patients with glioma in general. The gender distribution was nearly representative of the population as a whole, but with slightly more men than women enrolled than present in the general population of these patients. While these CNS tumors are nearly twice as common in white patients than black patients, black patients were under represented in this sample. |

1. Louis DN, Perry A, Wesseling P, Brat DJ, Cree IA, Figarella-Branger D, et al. The 2021 WHO classification of tumors of the central nervous system: a summary. Neuro-oncology. 2021;23(8):1231-51.

2. Wen PY, Macdonald DR, Reardon DA, Cloughesy TF, Sorensen AG, Galanis E, et al. Updated response assessment criteria for high-grade gliomas: response assessment in neuro-oncology working group. J Clin Oncol. 2010;28(11):1963-72.

3. Ostrom QT, Gittleman H, Xu J, Kromer C, Wolinsky Y, Kruchko C, et al. CBTRUS Statistical Report: Primary Brain and Other Central Nervous System Tumors Diagnosed in the United States in 2009-2013. Neuro Oncol. 2016;18(suppl_5):v1-v75.

4. Board CNE. 2022.
